# National influenza surveillance systems in five European countries: a qualitative comparative framework based on WHO guidance

**DOI:** 10.1186/s12889-022-13433-0

**Published:** 2022-06-09

**Authors:** Thierry Rigoine de Fougerolles, Oliver Damm, Filippo Ansaldi, Maria Chironna, Pascal Crépey, Simon de Lusignan, Ian Gray, José Maria Guillen, George Kassianos, Anne Mosnier, Raul Ortiz de Lejarazu, Elena Pariani, Joan Puig-Barbera, Jörg Schelling, Francesca Trippi, Philippe Vanhems, Klaus Wahle, John Watkins, Anvar Rasuli, Olivier Vitoux, Hélène Bricout

**Affiliations:** 1 CVA, Paris, France; 2 Sanofi-Aventis Deutschland GmbH, Berlin, Germany; 3 University of Genoa, Genoa, Italy; 4 Department of Interdisciplinary Medicine – Hygiene Section, University of Bari, Bari, Italy; 5 Université de Rennes, EHESP, CNRS, Inserm, Arènes - UMR 6051, RSMS – U 1309, Rennes, France; 6 University of Oxford, Oxford, UK; 7 Royal College of General Practitioners, London, UK; 8 Sanofi Pasteur, Reading, UK; 9 Sanofi Pasteur, Madrid, Spain; 10 Open Rome, Paris, France; 11 Centro Nacional de Gripe, Valladolid, Spain; 12 Department of Biomedical Sciences for Health, University of Milan, Milan, Italy; 13 Fisabio, Valencia, Spain; 14 Ludwig Maximilians University, Munich, Germany; 15 Sanofi Pasteur, Milan, Italy; 16 CIRI, Centre International de Recherche en Infectiologie, (Team (PHE3ID), Univ Lyon, Inserm, U1111, Université Claude Bernard Lyon 1, CNRS, UMR5308, ENS de Lyon, F-69007 Lyon, France; 17 Hospices Civils de Lyon and Hospices Civils de Lyon (HCL), Lyon, France; 18 Westfälische Wilhelms-Universität, Munich, Germany; 19 Cardiff University, Cardiff, Wales; 20 Sanofi Pasteur, Lyon, France

**Keywords:** Comparison, Europe, Influenza, Qualitative evaluation framework, Surveillance systems

## Abstract

**Background:**

Influenza surveillance systems vary widely between countries and there is no framework to evaluate national surveillance systems in terms of data generation and dissemination. This study aimed to develop and test a comparative framework for European influenza surveillance.

**Methods:**

Surveillance systems were evaluated qualitatively in five European countries (France, Germany, Italy, Spain, and the United Kingdom) by a panel of influenza experts and researchers from each country. Seven surveillance sub-systems were defined: non-medically attended community surveillance, virological surveillance, community surveillance, outbreak surveillance, primary care surveillance, hospital surveillance, mortality surveillance). These covered a total of 19 comparable outcomes of increasing severity, ranging from non-medically attended cases to deaths, which were evaluated using 5 comparison criteria based on WHO guidance (granularity, timing, representativeness, sampling strategy, communication) to produce a framework to compare the five countries.

**Results:**

France and the United Kingdom showed the widest range of surveillance sub-systems, particularly for hospital surveillance, followed by Germany, Spain, and Italy. In all countries, virological, primary care and hospital surveillance were well developed, but non-medically attended events, influenza cases in the community, outbreaks in closed settings and mortality estimates were not consistently reported or published. The framework also allowed the comparison of variations in data granularity, timing, representativeness, sampling strategy, and communication between countries. For data granularity, breakdown per risk condition were available in France and Spain, but not in the United Kingdom, Germany and Italy. For data communication, there were disparities in the timeliness and accessibility of surveillance data.

**Conclusions:**

This new framework can be used to compare influenza surveillance systems qualitatively between countries to allow the identification of structural differences as well as to evaluate adherence to WHO guidance. The framework may be adapted for other infectious respiratory diseases.

**Supplementary Information:**

The online version contains supplementary material available at 10.1186/s12889-022-13433-0.

## Background

Seasonal influenza represents a significant clinical and economic burden globally, with an attack rate estimated of 5–10% in adults and 20–30% in children [1] and up to 650,000 deaths globally and 72,000 deaths in Europe resulting from influenza-associated complications in all ages each year [2]. Although surveillance systems that continuously monitor the virology and epidemiology of seasonal influenza are now in place in most countries [3, 4], these are associated with varying degrees of success due to the unpredictable nature of influenza epidemics, the multiple data sources involved, and the varied degree of sophistication and funding in national surveillance systems [5]. The main purpose of such surveillance is to anticipate and protect the population and health systems from the threat of seasonal epidemics of influenza virus. The coronavirus infectious disease (COVID-19) pandemic caused by the severe acute respiratory syndrome coronavirus 2 (*SARS-CoV-2*) has highlighted the importance of surveillance and detection capabilities to mitigate and tackle the spread of infectious disease.

In its Global Influenza Strategy 2019–2030 [6], the World Health Organization (WHO) included influenza surveillance as part of its strategic objectives, as a key component to support public health policy making, feed influenza research, and anticipate pandemics .

Historically, influenza surveillance systems have been mainly focused on identifying influenza virus circulation patterns (antigenic drift and antigenic shift monitoring) and providing virology information to WHO Collaborating Centres. In 2009, the H1N1 pandemic provided a catalyst to improve the methods for measuring the overall impact of influenza including associated severe outcomes such as hospital admissions and deaths [7]. In developed countries, influenza has been monitored for several decades using sentinel networks where selected general practitioners (GPs) and hospitals collect swab samples from patients with influenza-like illness (ILI) or acute respiratory infection (ARI) in line with WHO guidance [8, 9]. Most advanced surveillance systems also use digital tools [10] and diverse statistical methods [11] to complement data from healthcare systems. In addition to data generation, the WHO encourages dissemination of data in a regular manner through consolidated and structured weekly and annual influenza surveillance reports [12–17], the data from which are often used in influenza public health awareness campaigns. Such continuous communication is particularly important for a seasonal disease such as influenza for which vaccine prevention exists. Public health agencies, as reliable public sources of data on influenza burden, play a key role in providing public information regarding the importance of protection against the influenza viruses. However, despite guidance and coordination efforts from the WHO through the Global Influenza Surveillance and Response System (GISRS) [18] and further coordination by the European Centre for Disease Protection and Control (ECDC) through the European Influenza Surveillance Network (EISN) [19] and Flu News Europe [4] (which use the European Surveillance System [TESSy] database), data generation and dissemination from influenza surveillance systems vary widely between countries. Guidance and tools to standardize, characterize and evaluate national influenza surveillance system were developed such as those from the United States Centers for Disease Control and Prevention [20, 21], and the handbook of methods and applications from the ECDC [22], in complement to the biennial publication of Influenza Surveillance Country Profiles in Europe [23].

The purpose of this qualitative study was to develop a comparative framework that capitalizes on the WHO guidance and provides countries with a platform for comparison of their influenza surveillance systems across a set of evaluation criteria. The aims were also to identify structural differences in the collection and dissemination of data on the burden of influenza, based on a comparative, qualitative evaluation of the influenza surveillance systems in place in five of the largest European countries (France, Germany, Italy, Spain, and the United Kingdom [UK]).

## Methods

Surveillance scope and comparative outcomes were first defined. Further criteria and sub-criteria were then defined to form a qualitative evaluation framework for influenza surveillance in France, Germany, Italy, Spain, and the UK to allow a qualitative comparison between countries. The evaluation was conducted between June 2019 and November 2020.

### Surveillance sub-systems and outcomes to be compared

The influenza surveillance sub-systems for which key outcomes were to be compared qualitatively were identified based on WHO guidance (virological surveillance, primary care surveillance and hospital surveillance) [8, 9] and the existing ECDC framework [23]. Four additional sub-systems were added: non-medically attended community surveillance [24], community surveillance, outbreak surveillance, and mortality surveillance (Table 1). These additional sub-systems considered the whole clinical spectrum of influenza disease and were based on surveillance tools used in the five countries and complemented by pan-European initiatives including the use of digital tools for influenza monitoring in the Influenzanet network [25] and excess mortality modelling as part of the EuroMOMO project [26]. The resulting set of 7 surveillance sub-systems was discussed and agreed by co-authors, including Sanofi Pasteur and a panel of influenza surveillance experts and researchers from each country (Table 2). Experts were chosen based on their experience and knowledge of influenza surveillance systems in the five countries. A roundtable event was held in October 2019 at which approximately 30 experts (including the 21 authors) reviewed, discussed, and adjusted the framework and the results of the comparative analyses. The group of experts included epidemiologists, virologists, general practitioners, public health researchers and pharmaceutical industry medical experts from the five countries included in the analysis (ie, France, Germany, Italy, Spain, and the UK). The selection of the authors from the group of experts was based on their agreement to fulfil the International Committee of Medical Journal Editors (ICMJE) criteria for authorship.

**Table 1:** Influenza surveillance sub-systems and comparative outcomes

Surveillance sub-system	Outcome
1. Non-medically attended community surveillance	1.1. ARI/ILI cases and/or incidence rates
	1.2. Proportion of ARI/ILI cases attending a physician^a^
2. Virological surveillance	2.1. ARI/ILI specimens for virus typing & subtyping
	2.2. ARI/ILI specimens for virus genome sequencing
	2.3. ARI/ILI specimens for antiviral drug resistance
3. Community surveillance	3.1. Notified biologically/laboratory-confirmed cases
4. Outbreak surveillance	4.1. ARI/ILI outbreaks in closed settings
	4.2. Biologically/laboratory-confirmed outbreaks in closed settings
5. Primary care surveillance	5.1. ARI/ILI GP visits and/or incidence rates
	5.2. Biologically/laboratory-confirmed GP visits and/or incidence rates
	5.3. Influenza-associated excess GP visits
	5.4. Influenza-associated excess work-loss cases
6. Hospital surveillance	6.1. ILI or biologically/laboratory-confirmed Emergency Department visits
	6.2. SARI/ILI hospital admissions
	6.3. Biologically/laboratory-confirmed hospital admissions
	6.4. Influenza-associated excess hospital admissions
	6.5. Biologically/laboratory-confirmed influenza ICU admissions
7. Mortality surveillance	7.1 Diagnosed or biologically/laboratory-confirmed influenza deaths
	7.2. Influenza-associated excess deaths

*GP* general practitioner, *ICU* intensive care unit, *ILI* influenza-like illness, *(S) ARI* (severe) acute respiratory illness.

^a^Includes self-reported (eg, through community surveys or phone advice lines) attendance at a healthcare setting.

**Table 2:** Sources of information by country

		France	Germany	Italy	Spain	United Kingdom	Total
Interviews	Sanofi	4	5	3	3	3	18
	External experts	6	3	3	5	3	20
Roundtable	Sanofi	1	1	1	1	1	5
	External experts	5	2	3	2	3	15
Total		16	11	10	11	10	58

Data are number of people.

Together these 7 surveillance sub-systems created a scope of case reporting reflecting increasing case severity, from non-medically attended cases in the community to fatal cases. For these 7 sub-systems, a total of 19 outcomes were defined to be used as a basis for comparison between countries. The 7 surveillance sub-systems and their associated outcomes are listed in Table 1.

### Selection of comparison criteria and definition of sub-criteria

A further 5 criteria were developed to qualitatively evaluate each of the surveillance sub-systems and outcomes described above, based on three criteria from the WHO manual for estimating disease burden associated with seasonal influenza [9] (completeness, representativeness, and accuracy of case count; a fourth criterion in the WHO manual assessing the potential for bias was excluded due to the lack of publicly available evidence to evaluate it). Two criteria were reworded to best fit the underlying sub-criteria, completeness into timing and accuracy into sampling strategy. Two further criteria were included to align with the WHO global epidemiological surveillance standards for influenza [8]: data granularity (to include how data are stratified by age, strain, chronic condition, and influenza vaccination status) and data dissemination (to include how data are shared by relevant Health Authorities through weekly and annual reports in line with WHO guidance) [6]. This resulted in the following 5 qualitative comparison criteria and associated sub-criteria for the evaluation of data for each of the surveillance sub-systems (for further details of the criteria, sub-criteria, and associated WHO guidance see Table 3):

- Granularity: stratification of data by age, gender and risk condition, insights on the geographical location, breakdown per virus type, subtype, and lineage, further patient details such as the severity of the infection (e.g. need for mechanical ventilation), the treatment administered, and vaccination status if available.
- Timing: continuity and frequency of data collection over a defined period.
- Representativeness: geographical and population representativeness based on the territory and population groups covered by sentinel systems and other surveillance components. For sentinel schemes, the number and proportion of facilities involved.
- Sampling strategy: based on the surveillance type and syndromic surveillance criteria used (ILI or ARI), the strategy to collect specimens and the type of laboratory test performed.
- Communication: availability in weekly and annual surveillance reports in a timely and open access manner for further use by researchers, the media, and other stakeholders.

**Table 3:** Criteria and sub-criteria (primary care surveillance example)

Criteria	Sub-criteria	WHO Guidance^a^
Granularity	Age group	Recommended as a minimum: 0–1, 2–4, 5–14, 15–49, 50–64, 65+ years and ideally additional age strata for under 2 years including 0 to < 6 months, 6 month to < 1 year, 1 to < 2 years
	Gender	Where possible data should be extracted by gender
	Risk condition	Recommended as a minimum: pregnancy status & presence of chronic pre-existing medical illness(es): chronic respiratory disease, asthma, diabetes, chronic cardiac disease, chronic neurological or neuromuscular disease, haematological disorders, immunodeficiency (including Human Immunodeficiency Virus)
	Location	Considered as essential, especially for burden estimation for a given area based on data from sentinel sites
	Virology	Types and subtypes of viruses detected during the week
	Severity	Additional data to consider: signs and symptoms of illness & patient outcome (death, survival)
	Treatment	Exposure to influenza antiviral drugs during the last 14 days? If yes, name of antiviral
	Vaccination status	Additional data to consider: Seasonal influenza vaccination status and date of administration
Timing	Frequency	Epidemiological and virological data collected from the sentinel sites should be reported to the national health authorities on a weekly basis
	Time period	In temperate climate zones where influenza seasonality is well understood, data collection and reporting should occur at a minimum during the known influenza season and for a short period preceding and following the season
Representativeness	Geographical representativeness	National - sentinel sites should include patients that will appropriately represent the population
	Population representativeness	The population served by the sentinel site should be representative of the target age and socioeconomic groups in the population under surveillance
	Number of settings	There is no ideal number of sentinel sites in a country. Start small with one or a few sentinel sites and only expand if these function well. Minimal information that should be presented in the weekly report includes number of sentinel sites reporting
	Proportion of facilities	Ideally the following analyses can be presented in an annual report: data from the monitoring of the system: proportion of sentinel sites reporting weekly to the national level; and if feasible, the proportion of sentinel sites regularly submitting specimens for laboratory testing
Sampling strategy	Surveillance type	Sentinel surveillance
	ARI/ILI definition	An acute respiratory infection with fever ≥38 °C and cough with onset within the last 10 days
	Sampling	A systematic approach to case selection that does not leave the choice of cases to test or gather data from up to healthcare providers (other than to determine that the case meets the definition), and that covers different times of the day and different days of the week is likely to be the most pragmatic, while providing reasonably representative data
	Test type	Reverse transcriptase-polymerase chain reaction (RT-PCR) is the most sensitive method for detecting influenza virus and is the recommended influenza surveillance assay for laboratories
Communication	In annual report	Yearly surveillance report with surveillance and risk factor data should be produced
	In weekly report	Weekly surveillance reports should be produced and made accessible to relevant partners
	Delay in release	Reports should provide timely information on influenza activity and types of influenza viruses circulating
	Data can be extracted	Whenever feasible, such reports should be available to the public on the national surveillance website

*ARI* acute respiratory illness, *ILI* influenza-like illness, *RT-PCR* reverse transcriptase-polymerase chain reaction.

^a^From WHO global epidemiological surveillance standards for influenza [8] and WHO manual for estimating disease burden associated with seasonal influenza [9]

Further information is included in the additional file.

### Data collection

Based on the qualitative comparative framework, the following 3-step process was used to ensure the quality and accuracy of information collected from the five countries:

1. The framework was completed using publicly available information from national influenza surveillance methodologies, weekly and annual reports of influenza surveillance [12–16].
2. Telephone or face-to-face interviews of approximately 1 hour were conducted with 2–5 influenza experts and researchers in each country. The interviewees included but were not limited to the co-authors and were selected based on their clinical experience of influenza and their expertise of national surveillance systems (see earlier).
3. The data collected from the framework were discussed with the panel of influenza experts and researchers from the five countries at the roundtable event described earlier. This allowed the structure of the framework to be further refined and for each country’s input to be adjusted where necessary. Details on the number of experts interviewed and participants at the roundtable for each country are presented in Table 2.

## Results

Using the qualitative framework described, detailed data for the comparison criteria are presented for each surveillance sub-system (non-medically attended community surveillance, virological surveillance, community surveillance, outbreak surveillance, primary care surveillance, hospital surveillance, and mortality surveillance) in the additional file. A synthetic overview of these data for each country is presented in Table 4.

**Table 4:** Overview of the comparative framework of influenza surveillance systems

		France	Germany	Italy	Spain	United Kingdom (England & Wales)	
Surveillance sub-system	Outcome	Santé publique France	RKI Arbeitsgemeinschaft für Influenza	Istituto Superiore di Sanita	Instituto de Salud Carlos III	Public Health England	Public Health Wales
1. Non-medically attended community surveillance	1.1. ARI/ILI cases and/or incidence rates	Web-survey (Grippe.net) & Phone advice line (SOS Médecins)	Web-survey (GrippeWeb)	Web-survey (InfluWeb)	Web-survey (GripeNet)	Web-survey (Flusurvey.net), web queries (Fludetector) & Phone advice line (NHS 111 Calls)	Web-survey (Flusurvey.net), Phone advice line (NHS Direct Wales)
	1.2. Proportion of ARI/ILI cases attending a physician						
2. Virological surveillance	2.1. ARI / ILI specimens for virus typing & subtyping	1 WHO NIC with 3 labs (including 1 WHO H5 and isolates from Réseau Sentinelles & RENAL)	1 WHO NIC (NRZ) (including isolates from regional labs)	1 WHO NIC & InfluNet-Vir (20 labs)	3 WHO NIC & sentinel labs (ReLEG)	1 WHO CC, 1 WHO ERL, NIC & Sentinel labs (Respiratory Datamart)	Public Health Wales Microbiology services
	2.2. ARI / ILI specimens for virus genome sequencing			1 WHO NIC	3 WHO NIC	1 WHO CC, 1 WHO ERL & 1 NIC	
	2.3. ARI / ILI specimens for antiviral drug resistance						
3. Community surveillance	3.1.. Notified biologically/laboratory-confirmed cases	Sentinel labs (RENAL)	Mandatory notification (IfSG)	None or unpublished	Sentinel Labs (ReLEG)	Sentinel labs (Respiratory Datamart)	
4. Outbreak surveillance	4.1. ARI/ILI outbreaks in closed settings	Care homes (Ehpad)	None	Unpublished	Schools, care homes, medical settings	Schools (MOSA) & care homes (PHE surveillance scheme)	PH Wales Health Protection team
	4.2. Biologically/laboratory-confirmed outbreaks in closed settings		Mandatory notification (IfSG)	Unpublished			
5. Primary care surveillance	5.1. ARI/ILI GP visits and/or incidence rates	1450 ARI & ILI Sentinel GPs (Réseau Sentinelles) & Phone advice line (SOS Médecins)	830 ARI sentinel GPs (AGI-Sentinel)	1000 ILI sentinel GPs (Influnet-Epi & Vir)	770 Sentinel GPs & mandatory notification (EDOs)	200–500 ILI sentinel GPs (RCGP RSC)	GP Sentinel Surveillance of Infections Scheme
	5.2. Biologically/laboratory-confirmed GP visits and/or incidence rates	300 ARI & ILI Sentinel GPs			770 Sentinel GPs (SVGE)		
	5.3. Influenza-associated excess GP visits	None	Statistical modelling (RKI model)	None	None	None	None
	5.4. Influenza-associated excess work loss cases	None		None	None	None	None
6. Hospital surveillance	6.1. ILI or biologically/laboratory-confirmed emergency department visits	~ 700 ARI sentinel hospitals (OSCOUR)	None	Unpublished	None	25 ARI sentinel hospitals (EDSSS)	ARI Sentinel hospitals
	6.2. SARI/ILI hospital admissions		~ 73 SARI sentinel hospitals (ICOSARI)	Unpublished	ARI (Chosp) & 100 SARI (CGHCG) sentinel hospitals	44 ARI sentinel hospitals (USISS)	
	6.3. Biologically/laboratory-confirmed hospital admissions		Mandatory notification (IfSG)	Unpublished			
	6.4. Influenza-associated excess hospital admissions	None	Statistical modelling (RKI model)	None	None	None	None
	6.5 Biologically/laboratory-confirmed ICU admissions	~ 192 SARI Sentinel hospitals (SpF)	None	SARI sentinel hospitals	ARI (Chosp) & 100 SARI (CGHCG) sentinel hospitals	All ICUs (USISS mandatory)	PH Wales Health Protection team
7. Mortality surveillance	7.1 Diagnosed or biologically/laboratory-confirmed influenza deaths	~ 192 SARI Sentinel hospitals (SpF) & death registries (SurSaUD & CépiDc)	Mandatory notification (IfSG)	SARI sentinel hospitals	ARI (Chosp) & 100 SARI (CGHCG) sentinel hospitals	All ICUs (USISS mandatory)	PH Wales Health Protection team
	7.2. Influenza-associated excess deaths	Statistical modelling (SurSaUD, CépiDc, SpF algorithm & FluMOMO)	Statistical modelling (RKI algorithm + FluMOMO in Berlin & Hesse)	Statistical modelling (Sismg EuroMOMO)	Statistical modelling (EuroMOMO)	Statistical modelling (FluMOMO)	Statistical modelling (EuroMOMO)

None = there are no surveillance tools covering this outcome; unpublished = these outcomes are covered by surveillance tools but the data are not published in the weekly or annual reports.

*AGI* Arbeitsgemeinschaft Influenza (influenza working group), *CépiDC* Centre d’épidémiologie sur les causes médicales de décès (epidemiological center on medical causes of deaths), *CGHCG* Casos graves hospitalizados confirmados de gripe (severe hospitalised influenza cases), *EDO* Enfermedades de Declaración Obligatoria (disease under mandatory notification), *EDSSS* Emergency Department Syndromic Surveillance System, *Ehpad* Etablissement d’Hébergement pour Personnes Âgées Dépendantes (nursing home), *GP* general practitioner, *ICOSARI* Hospital surveillance system for severe acute respiratory infections, *IfSG* Infektionsschutzgesetz (law on protection against infectious diseases), *MOMO* Mortality Monitoring, *MOSA* Medical Officers of Schools Association, *NHS* National Health System, *NIC* National Influenza Center, *NRZ* Nationales Referenzzentrum für Influenzaviren, *OSCOUR* Organisation de la Surveillance COordonnée des Urgences (organisation for coordinated emergency department surveillance), *PH* public health, *PHE* Public Health England, *RCGP RSC* Royal College of General Practitioners Research and Surveillance Centre, *ReLEG* Red de Laboratorios de Gripe en España (Spanish network of laboratories for influenza), *RENAL* Réseau national des laboratoires hospitaliers (network of hospital laboratories), *RKI* Robert Koch Institute, *(S) ARI* (severe) acute respiratory illness, *Sismg* Sistema di Sorveglianza della Mortalità Giornaliera (daily mortality surveillance system); Santé publique France, *SurSaUD* Surveillance Sanitaire des Urgences et des Décès (public health surveillance of emergencies and deaths), *SVGE* Sistema Centinela de Vigilancia de Gripe en España (Spanish influenza surveillance sentinel system), *USISS* UK Severe Influenza Surveillance System, *WHO CC* World Health Organization Collaborating Centre, *WHO ERL* WHO Essential Regulatory Laboratory.

### Overview of the comparative framework

France and the UK (England and Wales) had the widest scope across all 7 surveillance systems, monitoring 16 out of 19 outcomes, thanks to the breadth of the outcomes covered in hospital surveillance. All of the five countries included in the analysis met the basic WHO requirements, with well-established networks of laboratories and sentinel schemes for primary care and hospital surveillance.

For virological surveillance (surveillance sub-system 2: Table 4), each country had one or more WHO National Influenza Centers (NIC) to perform typing and subtyping of strains, sequence the whole genome, and evaluate the antiviral drug resistance of the influenza samples provided by sentinel schemes.

Primary care surveillance (surveillance sub-system 5: Table 4) was also well developed with all countries having sentinel GP systems (including pediatricians) reporting and testing patients with ARI and/or ILI symptoms. No data were collected for influenza-associated excess GP visits or absenteeism except for Germany where statistical modelling was in place for primary care surveillance.

Hospital surveillance (surveillance sub-system 6: Table 4) was also implemented in all countries, but with differences in terms of breadth of the scope covered. In France, England and Wales, surveillance systems routinely monitored ILI emergency department (ED) visits. German surveillance included statistical modelling for influenza-associated hospitalizations, but did not cover ED visits and Intensive Care Unit (ICU) admissions. In Spain, hospital surveillance included all outcomes except ED visits and statistical modelling for influenza related hospitalizations. In Italy hospital surveillance was focused on the most severe outcomes, reporting only data on ICU admissions and with no published data on other outcomes from this sub system.

All countries had non-medically attended community surveillance and mortality surveillance systems in place (although in Spain web survey data had not been published since 2016) (surveillance sub-systems 1 and 7: Table 4). These were coordinated by the InfluenzaNet and EuroMOMO European initiatives, respectively. Worth noting is that Italy and Spain modelled excess mortality for all causes using EuroMOMO model, whereas France, England and two German states (Berlin and Hesse) produced excess mortality estimates attributable to influenza using the FluMOMO model. However, the Robert Koch Institute uses its own model to estimate influenza attributable excess mortality estimates.

There were significant differences across the five countries for community surveillance and outbreak surveillance (surveillance sub-systems 3 and 4: Table 4). Biologically or laboratory-confirmed cases were notified by sentinel laboratories in France, Spain and the UK and suspected or laboratory-confirmed influenza outbreaks in some closed settings (e.g. schools or nursing homes) were published on a weekly basis. Germany mandated notification for any community-notified laboratory-confirmed cases and outbreaks in closed settings. In Italy, data for mandatory notification were either not collected (notified laboratory-confirmed cases) or not published by the health authorities (outbreaks in closed settings).

No country performed the collection of data on complications and events after hospitalization, i.e. the pathway of care.

### Performance across the 5 comparison criteria

The main findings for the qualitative comparison criteria (granularity, timing, representativeness, sampling strategy, and communication [as defined in Table 3]) for each surveillance sub-system are described below. Further details are included in the additional file.

Data granularity was below WHO standards in most countries, especially regarding the stratification by risk condition (defined in Table 3), which was available in France and Spain for some outcomes but not in the UK, Germany, and Italy (see additional file for details). Except in the UK and some regions of Spain, the absence of information on influenza immunization status was also common; this was particularly apparent in France, Italy, and Germany due to a lack of both a vaccination registry and standardized vaccination software tools in GP and pediatric practices.

The timing of the data was well aligned between countries, with surveillance starting usually at Week 40 (end of September) and ending at Week 20 (mid-May) the following year and most outcomes provided on a weekly basis.

The representativeness of the data would be expected to be affected by major differences in the proportion of the population covered by sentinel surveillance by GPs and pediatricians. Coverage of the territory and population also varies for influenza hospital surveillance, with 60 to 70% of the French population and about 50% of the Spanish population but only 6% of the German population covered by national hospital influenza surveillance schemes. Data were not available for Italy and the UK at the time of the study.

The sampling strategy was overall aligned across countries although there were nuances in terms of syndromic criteria (ARI, ILI, or severe ARI) used in hospital surveillance and the swabbing strategy applied by sentinel GPs and pediatricians. However, the use of reverse transcriptase polymerase chain reaction (RT-PCR) testing was the main standard for virological analysis coordinated by influenza national reference centres in each country.

Communication of data was inconsistent between the five countries. In the UK, France, Germany, and Spain, consolidated weekly and annual reports were provided in a timely manner that were easily accessible and manageable, except national excess mortality estimates in Germany which only were available with a delay of one year. In contrast, in Italy influenza surveillance data were issued in multiple formats, in a range of reports, and via various web pages. All five countries shared weekly surveillance data, including outside the usual epidemic period, on influenza circulation and virology with the WHO and ECDC.

### Country-specific observations

#### France

In general, a wide scope of surveillance sub-systems was used, especially for virological, outbreak, primary and secondary care surveillance. Multiple tools were used for community surveillance, but the use of modelling for influenza-associated events was limited, except for estimating mortality. There was a high level of data granularity, especially for risk conditions, and the representativeness of the data was good due to extensive sentinel networks in hospital surveillance. This hospital surveillance included alert thresholds regarding the number of patients admitted to EDs that triggered different responses based on hospital-specific strategies. Insights on post-hospitalization events (e.g. the impact on frailty/autonomy) and effect of influenza on the economy and healthcare systems were limited.

#### Germany

Overall, a large number of surveillance outcomes was covered (including excess consultations and absenteeism), but ED visits were not included in the surveillance and there was very limited information available for ICU admissions. Excess mortality estimates did not include a break down by age, and the European FluMOMO model [26] was only used in two federal states (Berlin and Hesse).

There was a lack of granularity for risk condition and vaccination status and limited insights into complications and treatment.

Data accuracy in hospital surveillance was affected by a wide syndromic surveillance scheme that included influenza as well as other acute lower respiratory tract infections, and by excess hospitalization estimates relying on data shared by primary care GPs that likely leads to an underestimation of cases. The communication of some surveillance data graphically rather than in tabular format impedes their use in further research.

#### Italy

There was no systematic notification of influenza cases in the community, whilst outcomes related to ARI / ILI outbreaks in closed settings, ED visits and hospital admissions were collected but not disseminated publicly. There was a lack of granularity by age and risk condition, the latter not being collected by a form used for GP sentinel surveillance and information on the severity of influenza among cases admitted in ICU was limited.

#### Spain

Virological, outbreak, primary care, hospital and mortality surveillance are all covered, whilst non-medically attended community ILI surveillance web survey data had been performed for the previous 8 influenza seasons [25, 27] but not published since 2016. Cases of ILI reported by all GPs through mandatory notification Enfermedades de Declaración Obligatoria (EDO) were not included in the annual report as ILI incidence data from sentinel GPs was preferred. For hospital surveillance, there was no information on ED visits due to influenza. Despite the standardized influenza surveillance methods across regions coordinated by the Spanish Institute of Public Health [28], regional disparities were observed in terms of data granularity and the proportion of the population covered by sentinel networks, and Galicia, Murcia, and Aragόn did not provide data on ILI as they did not have GP sentinel surveillance networks in place at the time of the research [29].

#### United Kingdom

There was a large variety of surveillance tools used to cover all sub-systems and a high level of data granularity, except for data on risk condition that were not made public, except in research articles. However, as for the other countries, the surveillance did not extend to post-hospital follow-up of discharged patients, i.e. the pathway of care.

## Discussion

The qualitative framework that we describe is more detailed than the resources available via the WHO and could be used as a standardised tool to systematically evaluate a country’s influenza infrastructure against WHO standards and other countries. The development and evaluation of this new framework allows stakeholders to use 7 sub-systems and 19 outcomes as well as 5 comparison criteria to identify and develop areas for possible improvement in influenza surveillance in terms of data collection and dissemination. The framework was largely developed before the *SARS-CoV-2* pandemic has since been applied to compare *SARS-CoV-2* and seasonal influenza surveillance in the same five European countries that are included in the present evaluation (ie, France, Germany, Italy, Spain, and UK) [30]. Additionally, the framework has been used to compare influenza surveillance systems in Asia-Pacific countries (Australia, China, and Malaysia) [31].

The five comparison criteria (granularity, timing, representativeness, sampling strategy, and communication) and sub-criteria also provide a means to qualitatively evaluate adherence to WHO standards and to objectively assess and compare the subsystems between countries. The compilation of these criteria is broader than has been reviewed in the literature, which focuses on granularity and timing [7, 32] or sampling strategy (i.e. harmonization of case definitions) [33]. Although updates on guidance related to influenza surveillance are issued regularly by the WHO as part of the GISRS [18] and Joint External Evaluations are performed, there are no explicit mechanisms for national public health agencies to perform comparative assessments of their systems to drive improvements in surveillance. A review of influenza surveillance systems in 2014 focused on lower and middle income countries [34] and a standard country surveillance profile regularly covers European Union Member States [23] but to our knowledge there is no available tool allowing universal comparisons of the current breadth of influenza surveillance systems using a structured list of criteria based on WHO guidance.

The qualitative variability of the influenza surveillance systems used by the 5 Western European countries included in our study was high. This is a direct result of the different sources used to collect the data in the different countries, the health system infrastructure, the health pathway, and in some cases the lack of available information. In the first subsystem, the use of digital tools for monitoring the incidence of non-medically attended ARI/ILI symptoms in the community was not always in place despite their relevance for the early detection of the seasonal influenza intensity and the understanding of the overall burden of influenza beyond patients attending medical facilities [24, 35]. In the second sub-system, virological surveillance, all five countries had WHO NICs contributing to the GISRS in line with WHO basic requirements, relying on laboratory-confirmed influenza samples collected through sentinel and/or non-sentinel sources. In the third sub-system, community surveillance, biologically and/or laboratory-confirmed influenza cases had to be notified only in Germany, whereas only sentinel laboratories notified such cases in France, Spain and the UK. In Italy, there was no such notification system nor were data published. For sub-system four, biologically or laboratory-confirmed outbreaks in settings such as nursing homes and long-term care facilities were not always well captured or published, despite a high death toll in the vulnerable elderly population due to influenza viruses [36]*. *Primary care surveillance through GP or paediatrician sentinel schemes (the fifth subsystem) was the most established across the five countries, and these data were well communicated at both the European and country level. Hospital surveillance, the sixth subsystem, is key to understanding severe influenza outcomes, but was inconsistent across the five countries despite WHO guidance being in place for over a decade [7]*. *In the seventh subsystem, mortality surveillance was complemented by all-cause excess mortality monitoring through the EuroMOMO project in Spain and Italy [26], but the FluMOMO model to estimate the share attributable to seasonal influenza was used in France and some parts of the UK (England only) and Germany (Berlin and Hesse) [37, 38]*.*

Influenza surveillance data play a key role to inform populations of the possible threats of seasonal influenza, to feed research to improve the understanding of the disease, and to inform policy-making through impact and health economics assessments of vaccination programmes and non-pharmaceutical interventions [39, 40]. The influenza vaccine coverage rate (VCR) in Organisation for Economic Cooperation and Development (OECD) countries has declined from 49 to 42% between 2007 and 2017 in adults aged 65 years and above [41]. A recent evaluation of four countries with high-performing influenza vaccination programs showed that a well-documented burden of influenza captured by robust surveillance systems and a high disease awareness among the lay public through communication from multiple stakeholders were key components to increasing VCR [42].

Furthermore, the value of well-established routine influenza surveillance systems that have been re-purposed for pandemic surveillance [43] could be further explored in light of the lessons learnt from the *SARS-CoV-2* pandemic. A comparison of SARS-CoV-2 and influenza surveillance in France, Germany, Italy, Spain, and UK has been published separately [30]*.* As has been evidenced during the *SARS-CoV-2* pandemic, individual preventative behaviour can also be important in the control of the spread of disease, and better awareness of the burden of influenza disease is likely to play a role in such behaviour [39, 40]*.* In the countries we evaluated, the economic impact of influenza (except for the assessment of absenteeism in Germany) and the disruption caused by influenza to the healthcare system in the winter are not routinely covered as part of the surveillance, nor is the pathway of care. Using economic impact and healthcare indicators could also prove valuable not only to better understand disease impact but also to better calibrate the efforts in terms of prevention and pandemic preparedness, and the WHO has issued a manual for estimating the economic burden of seasonal influenza [44] but which is yet to be included in the mandate of national surveillance systems.

The strength of our study is the development of a standardized, complete and enhanced framework to evaluate and compare influenza surveillance systems in different countries. Furthermore, the analysis was ambitious in scope and the data included in the additional file provide and in-depth assessment of 5 large European countries using the new framework. The data we describe are useful for routine influenza surveillance systems but also are adaptable for other emerging diseases, e.g. for the improvement of systems and allocation of healthcare resources, modelling, predictions, seasonality, and interactions with other diseases surveyed. Lastly, this framework may also be used to highlight strengths and identify gaps in existing systems that could be adapted in the surveillance of other circulating infectious respiratory pathogens such as respiratory syncytial virus or the newly emerged Omicron variant of *SARS-CoV-2* [30]. It is not the intention of this framework, however, to determine whether certain data sets are considered to be more important or meaningful than others nor how the data should be used for research or communication purposes.

Our study has a number of limitations. First, it was developed using 5 European countries with mature influenza surveillance systems and remains to be tested in countries with less advanced influenza surveillance systems. Second, the analysis was largely conducted before the *SARS-CoV-2* pandemic, since which time some influenza surveillance systems have been repurposed and synergies identified for influenza and *SARS-CoV-2* surveillance; these have been described in a separate analysis [30]. Third, the decentralized nature of the healthcare systems in some countries, such as Spain and Italy, or the different systems across the nations of the UK, may require the assessment to also be performed at a more granular level. Fourth, the exclusion of the assessment of potential for bias from the comparison criteria was necessary due to the limited public availability of information, although access may be better for public health agencies. Fifth, the framework does not cover all aspects that could lead to substantial improvements of influenza surveillance [45]. For instance, an evaluation of testing capacity and non-sentinel strategies, case definitions, swabbing protocols, compliance of healthcare professionals’ with guidelines in reporting data, the role of financial compensation for data reporting, or a comparison of paper-based or electronic reporting systems could enhance the analysis. Also, the FAIR principles (findable, accessible, interoperable, and reusable) [46] and the requirement for sentinel systems to adapt in the face of situations such as the recent *SARS-CoV-2* pandemic could be considered*.*

## Conclusions

In conclusion, we developed and tested a qualitative comparative framework to evaluate influenza surveillance systems in five countries, adapted from WHO guidance, which has identified structural differences between countries. This qualitative framework could encourage the adaptation and standardization of surveillance systems to improve the allocation of healthcare resources, and to further characterize the influenza burden. Following the *SARS-CoV-2* pandemic and the emergence of variants, including the newly emerged Omicron variant, we believe that the conclusions of this work might help move toward a more integrated, coordinated, and homogeneous European surveillance model for respiratory infectious diseases based on the systems currently in place for influenza.

## Supplementary Information

**Additional file 1.**

## Data Availability

All data generated or analysed during this study are included in this published article and its additional file.

## Abbreviations

AGI: Arbeitsgemeinschaft für Influenza (influenza working group)
CépiDC: Centre d’épidémiologie sur les causes médicales de décès (epidemiological center on medical causes of deaths)
CGHCG: Casos graves hospitalizados confirmados de gripe (severe hospitalised influenza cases)
EDO: Enfermedades de Declaración Obligatoria (disease under mandatory notification)
EDSSS: Emergency Department Syndromic Surveillance System
Ehpad: Etablissement d’Hébergement pour Personnes Âgées Dépendantes (nursing home)
GP: General practitioner
ICOSARI: Hospital surveillance system for severe acute respiratory infections
ICU: Intensive care unit
IfSG: Infektionsschutzgesetz (law on protection against infectious diseases)
ILI: Influenza-like illness
MOMO: Mortality Monitoring
MOSA: Medical Officers of Schools Association
NHS: National Health System
NIC: National Influenza Center
OSCOUR: Organisation de la Surveillance COordonnée des Urgences (organisation for coordinated emergency department surveillance)
PH: Public health
PHE: Public Health England
RCGP RSC: Royal College of General Practitioners Research and Surveillance Centre
RKI: Robert Koch Institute
RT-PCR: Reverse transcriptase-polymerase chain reaction
(S)ARI: (Severe) acute respiratory illness
Sismg: Sistema di Sorveglianza della Mortalità Giornaliera (daily mortality surveillance system)
SurSaUD: Surveillance Sanitaire des Urgences et des Décès (public health surveillance of emergencies and deaths)
SVGE: Sistema Centinela de Vigilancia de Gripe en España (Spanish influenza surveillance sentinel system)
USISS: UK Severe Influenza Surveillance System
