# Supplementary material for: National influenza surveillance systems in five European countries: a qualitative comparative framework based on WHO guidance
Source: BMC Public Health. 2022 Jun 9;22:1151. doi: 10.1186/s12889-022-13433-0 (PMC9178537; doi:10.1186/s12889-022-13433-0)
Supplement: Supplementary file 1 — Additional file 1. [file 12889_2022_13433_MOESM1_ESM.docx]

**ADDITIONAL FILE**

**National influenza surveillance systems in five European countries: a comparative framework based on WHO guidance**

Thierry Rigoine de Fougerolles^1^, Oliver Damm^2^, Filippo Ansaldi^3^, Maria Chironna^4^, Pascal Crépey^5^, Simon de Lusignan^6,7^, Ian Gray^8^, José Maria Guillen^9^, George Kassianos^7^, Anne Mosnier^10^, Raul Ortiz de Lejarazu^11^, Elena Pariani^12^, Joan Puig-Barbera^13^, Jörg Schelling^14^, Francesca Trippi^15^, Philippe Vanhems^16^, Klaus Wahle^17^, John Watkins^18^, Anvar Rasuli^19^, Olivier Vitoux^1^, Hélène Bricout^19^

^1^CVA, Paris, France; ^2^Sanofi-Aventis Deutschland GmbH, Berlin Germany; ^3^University of Genoa, Genoa, Italy; ^4^Department of Interdisciplinary Medicine – Hygiene Section, University of Bari, Bari, Italy; ^5^University of Rennes, École des Hautes Études en Santé Publique, REPERES, EA 7449, Rennes, France; ^6^University of Oxford, Oxford, UK; ^7^Royal College of General Practitioners, London, UK; ^8^Sanofi Pasteur, Reading, UK; ^9^Sanofi Pasteur, Madrid, Spain; ^10^Open Rome, Paris, France; ^11^Centro Nacional de Gripe, Valladolid, Spain; ^12^Department of Biomedical Sciences for Health, University of Milan, Milan, Italy; ^13^Fisabio, Valencia, Spain; ^14^Ludwig Maximilians University, Munich, Germany; ^15^Sanofi Pasteur, Italy; ^16^CIRI, Centre International de Recherche en Infectiologie, (Team (PHE3ID), Univ Lyon, Inserm, U1111, Université Claude Bernard Lyon 1, CNRS, UMR5308, ENS de Lyon, F-69007, Lyon, France and Hospices Civils de Lyon and Hospices Civils de Lyon (HCL), Lyon, France; ^17^Westfälische Wilhelms-Universität, Munich, Germany; ^18^Cardiff University, Cardiff, Wales; ^19^Sanofi Pasteur, Lyon, France

**1. Non-medically attended community surveillance**

|  |  | **France** | | | **Germany** | | | **Italy** | | | **Spain** | | | **United Kingdom** | **England** | | **Wales** | **Scotland** | **Northern Ireland** |
| --- | --- | --- | --- | --- | --- | --- | --- | --- | --- | --- | --- | --- | --- | --- | --- | --- | --- | --- | --- |
| **Surveillance tools** | | [**Web-survey**](https://www.grippenet.fr/) | **Web Queries** | **Medical advice line** | **Web-survey** | **Web Queries** | **Medical advice line** | **Web-survey** | **Web Queries** | **Medical advice line** | **Web-survey** | **Web Queries** | **Medical advice line** | [**Web-survey**](https://www.grippenet.fr/) | **Web Queries** | **Medical advice line** | **Medical advice line** | **Medical advice line** | **Medical advice line** |
|  |  | [Grippenet.fr](https://www.grippenet.fr/) | **NA** | **SOS Médecins** | [GrippeWeb.rki.de](https://grippeweb.rki.de/) | **NA** | **NA** | [Influweb.it](https://www.influweb.it/) | **NA** | **NA** | [Gripenet.es](https://www.gripenet.es/) | **NA** | **NA** | [Flusurvey.net](https://flusurvey.net/) | **FluDetector** | **NHS 111** | **NHS Direct Wales** | **NHS 24** | **NA** |
| **Outcome** | 1.1. ARI / ILI cases and/or incidence rates | ARI & ILI & general symptoms incidence rate | None | ARI & ILI calls & proportion over total calls | ARI & ILI cases & incidence rate | None | None | ARI & ILI cases & incidence rate | None | None | Results not published since 2016 | None | None | ILI incidence | ILI queries & incidence | ARI & ILI calls & incidence rate | Influenza-related calls & incidence rate | ARI & ILI calls & incidence rate | No |
|  | 1.2. Proportion of ARI/ILI cases attending a physician | Yes |  | Yes | Yes |  |  | Yes |  |  |  |  |  | Yes | No | No | No | No | No |
| **Granularity** | Age group | 0-90yo, per 5y group |  | Unpublished data | 0-4, 5-14, 15-34, 35-59, 60+ |  |  | 0-5, 6-12, 13-19, 20-39, 40-59, 60+ |  |  |  |  |  | <20, 20-44, 45+ | No | No | No | <1, 1-4, 5-14, 15-64, 65+ | No |
|  | Gender | Yes |  |  | Yes, but not used for stratification of results |  |  | Yes |  |  |  |  |  | Yes | No | No | No | No |  |
|  | Risk condition | Available |  |  | Yes, but not regularly published |  |  | Available |  |  |  |  |  | No | No | No | No | No |  |
|  | Location | No |  |  | Yes, but not used for stratification of results |  |  | Yes, per region |  |  |  |  |  | Yes (England, Scotland, Wales, NI) | No | No | No | No |  |
|  | Virology | NA |  |  | NA |  |  | NA |  |  |  |  |  | NA | NA | NA | NA | NA |  |
|  | Severity (symptoms, complications) | 20 ILI symptoms |  |  | Collected but unpublished |  |  | Collected but unpublished |  |  |  |  |  | 12 ILI symptoms | No | No | No | No |  |
|  | Treatment | Yes |  |  | No |  |  | No |  |  |  |  |  | No | No | No | No | No |  |
|  | Vaccination status | Available |  |  | Yes, but not regularly published |  |  | Yes |  |  |  |  |  | Available | No | No | No | No |  |
| **Timing** | Frequency | Weekly |  |  | Weekly |  |  | Weekly |  |  |  |  |  | Weekly | Daily, Weekly | Weekly | Weekly | Weekly |  |
|  | Time period | W41/46-W20 |  |  | All year long |  |  | W44-W16 |  |  |  |  |  | W42-W20 | All year long | All year long | All year long | All year long |  |
| **Representa-tiveness** | Geographical representativeness | National |  |  | National (397/401 districts) |  |  | National |  |  |  |  |  | UK | England | England (NHS 111) | Wales | Scotland |  |
|  | Population representativeness | ~9.5 per 100k |  |  | No info |  |  | 3.3 / 100k |  |  |  |  |  | 11.3 /100k | 10-15% sample of all queries | 100% | 100% | 100% |  |
|  | Number respondents | ~6k |  |  | ~7k (2018-2019 season) |  |  | ~2k-4.5k |  |  |  |  |  | ~4k (3.5k in England, 244 Scotland, 27 NI and 152 Wales) | NA | NA | NA | NA |  |
| **Sampling Strategy** | Surveillance type | Self-reported syndromic surveillance |  |  | Self-reported syndromic surveillance |  |  | Self-reported syndromic surveillance |  |  |  |  |  | Self-reported syndromic surveillance | Internet based syndromic surveillance | Remote health advice syndromic surveillance | Syndromic community surveillance | Syndromic community surveillance |  |
|  | Testing status | No |  |  | No |  |  | No |  |  |  |  |  | No | NA | NA | NA | NA |  |
| **Communication** | In annual report | Yes, specific annual report |  |  | Yes |  |  | Yes |  |  |  |  |  | No, specific report | Yes | Yes | Yes | Yes |  |
|  | In weekly report | No |  |  | Yes |  |  | No |  |  |  |  |  | Yes, sometimes | Yes | Limited | Yes | Limited |  |
|  | Delay in release (if any) | No |  |  | No |  |  | No |  |  |  |  |  | None | No | No | No | No |  |
|  | Data can be extracted | No |  |  | No |  |  | Yes |  |  |  |  |  | No | Yes | No | No | No |  |
|  |  | Data available & published | Unpublished data | None | No info | NA |  |  |  |  |  |  |  |  |  |  |  |  |  |

**2. Virological surveillance**

|  |  | **France** | | **Germany** | | **Italy** | | **Spain** | | **England** | | | **Wales** | **Scotland** | **Northern Ireland** |
| --- | --- | --- | --- | --- | --- | --- | --- | --- | --- | --- | --- | --- | --- | --- | --- |
| **Surveillance tools** | | **Sentinel laboratories** | **WHO NIC network** | **Sentinel laboratories** | **WHO NIC network** | **Sentinel laboratories** | **WHO NIC network** | **Sentinel laboratories** | **WHO NIC network** | **Sentinel laboratories** | **WHO NIC network** | **WHO CC network** | **Laboratory surveillance** | **Laboratory surveillance** | **Laboratory surveillance** |
|  |  | **Réseau Sentinelles & Réseau national des laboratoires hospitaliers (RENAL)** | **1 WHO NIC with 3 labs (including 1 H5 laboratory)** | **6 regional sentinel networks & laboratories** | **1 WHO NIC (NRZ), including national sentinel network** | **InfluNet-Vir** | **1 WHO NIC** | **Red de Laboratorios de Gripe en España (ReLEG)** | **3 WHO NIC** | **Respiratory DataMart system** | **1 WHO NIC** | **1 WHO CCR lab** | **Public Health Wales Microbiology Services** | **Electronic Communication of Surveillance in Scotland (ECOSS)** | **Regional Virus Laboratory (RVL)** |
| **Outcome** | 2.1. ARI / ILI specimens for virus typing & subtyping | Yes | Yes | Yes | Yes | Yes | Yes | Yes | Yes | Yes | Yes | Yes | Yes | Yes | Yes |
|  | 2.2. ARI / ILI specimens for virus genome sequencing | No | Yes | No | Yes | No | Yes | No | Yes | No | Yes | Yes | Yes | Yes | Yes |
|  | 2.3. ARI / ILI specimens for antiviral drug resistance | No | Yes | No | Yes | No | Yes | No | Yes | No | Yes | Yes | Yes | Yes | Yes |
| **Granularity** | Age group | Unpublished | Unpublished | Yes | Unpublished | No | No | No | No | No | No | No | >1, 1-4, 5-14, 15-24, 25-34, 35-44, 45-64, 65-74, 75+ | No | 0-4, 5-14, 15-64, 65+ |
|  | Gender | Unpublished | Unpublished | Yes | No | No | No | No | No | No | No | No | No | No | No |
|  | Risk condition | No | No | No | No | No | No | No | No | No | No | No | No | No | No |
|  | Location | No | No | Yes, per region | Yes, per region | Yes, per region | No | No | No | No | No | No | No | No | No |
|  | Virology | Flu virus type & sub-type, other viruses (RSV & others) | Flu virus type & sub-type, other viruses (RSV & others) | Flu virus type & sub-type, other viruses (RSV & others) | Flu virus type & sub-type, other viruses (RSV & others) | Flu virus type & sub-type | Flu virus type & sub-type, other viruses (RSV & others) | Flu virus type & sub-type | Flu virus type & sub-type, other viruses (RSV & others) | Flu virus type & sub-type, other viruses (RSV & others) | Flu virus type & sub-type, other viruses (RSV & others) | Flu virus type & sub-type, other viruses (RSV & others) | Flu virus type & sub-type, other viruses (RSV & others) | Yes virus type, sub-type | Flu virus type & sub-type, other viruses (RSV & others) |
|  | Severity (symptoms, complications) | No | No | Yes | No | No | No | No | No | No | No | No | No | No | No |
|  | Treatment | Unpublished | Unpublished | No | No | No | No | No | No | No | No | No | No | No | No |
|  | Vaccination status | Unpublished | Unpublished | Yes | No | No | No | No | No | No | No | No | No | No | No |
| **Timing** | Frequency | Weekly | Weekly | Weekly | Weekly | Weekly | Weekly | Weekly | Weekly | Weekly | Weekly | Weekly | Weekly | Weekly | Weekly |
|  | Time period | W40-W20 | W40-W20 | W40-W20 | All year long | W46-W17 | W40-W20 | W40-W20 | W40-W20 | W40-W20 | W40-W20 | W40-W20 | W40-W20 | Week 40-Week21 | All year long |
| **Representa-tiveness** | Geographical representativeness | National | National | Regional | National | National | National | National | National | National | National | National | Wales | Scotland | Northern Ireland |
|  | Population representativeness | No info | No info | From 6 federal states | No info | No info | No info | No info | No info | No info | No info | No info | 100% / mandatory | 100% / mandatory | 100% / mandatory |
|  | Number of settings | No info | 1 coordinating lab. (Institut Pasteur Paris) and 2 associated labs (Hospices Civils de Lyon & Institut Pasteur de Guyane) | 6 regional laboratories | 1 laboratory, Virological deprtament, Robert Koch Institute | No info | 1 laboratory, Istituto Superiore di Sanità | 20 laboratories, coordinated by the NIC Centro Nacional de Microbiología | 3 laboratories, Madrid, Barcelona, Vallodalid | England PHE and NHS laboratories | 1 laboratory, Public Health England, Respiratory Virus Unit, Colindale | 1 laboratory, Crick Worldwide Influenza Centre, London | 2 laboratories, Swansea and Cardiff | All HPS labs | All non-sentinel virology settings |
|  | Proportion of facilities | No info | NA | No info | NA | No info | NA | No info | NA | No info | NA | NA | No info | No info | No info |
| **Sampling Strategy** | Surveillance type | Active sentinel virological surveillance | Active sentinel virological surveillance | Active virological surveillance | Active & passive virological surveillance | Active sentinel virological surveillance | Active sentinel virological surveillance | Active & Passive virological surveillance | Active sentinel virological surveillance | Active & Passive virological surveillance | Active sentinel virological surveillance | Active sentinel virological surveillance | Passive systematic notification | Passive systematic notification | Non-sentinel virology surveillance |
|  | Sampling | From sentinel GPs (Réseau Sentinelle) & hospitals (RENAL) | From sentinel GPs (Réseau Sentinelle) & hospitals (RENAL) | From sentinel GPs | From sentinel GPs (AGI-Sentinel network) & others | From sentinel GPs, peads & hospitals | From sentinel GPs, peads & hospitals | From sentinel and non sentinel sources | From sentinel and non sentinel sources | From sentinel and non sentinel sources | From sentinel and non sentinel sources | From sentinel and non sentinel sources | From sentinel, non-sentinel GPs & all wards from hospitals | From non-sentinel sources, mostly hospitals | From GP practice, Hospitals, & others |
|  | Test type | RT-PCR & others (rapid point of care) | RT-PCR | RT-PCR | RT-PCR | RT-PCR | RT-PCR | RT-PCR | RT-PCR | RT-PCR | RT-PCR | RT-PCR | RT-PCR | RT-PCR & RPOCT | RT-PCR |
| **Communication** | In annual report | Yes | Yes | Yes | Yes | Yes | No | Yes | No | Yes | No | No | Yes | Yes | Yes |
|  | In weekly report | Yes | Yes | No | Yes | Yes | No | Yes | No | Yes | No | No | Yes | Yes | Yes |
|  | Delay in release | No | No | No | No | No | No | No | No | No | No | No | No | No | No |
|  | Data can be extracted | Yes | Yes | No | No | Yes | Yes | Yes | Yes | Yes | Yes | Yes | Yes | No | Yes |
|  |  | Data available & published | Unpublished data | None | No info | NA |  |  |  |  |  |  |  |  |  |

3. Community surveillance

|  |  | **France** | **Germany** | **Italy** | **Spain** | **England** | **Wales** | **Scotland** | **Northern Ireland** |
| --- | --- | --- | --- | --- | --- | --- | --- | --- | --- |
| **Surveillance tools** | | **Sentinel laboratories** | **Mandatory disease notification** | **Sentinel laboratories** | **Sentinel laboratories** | **Sentinel laboratories** | **Laboratory surveillance** | **Laboratory surveillance** | **Laboratory surveillance** |
|  |  | **Réseau national des laboratoires hospitaliers (RENAL)** | **IfSG obligation** | **NA** | **Red de Laboratorios de Gripe en España (ReLEG)** | **Respiratory DataMart system** | **Public Health Wales Microbiology Services** | **Electronic Communication of Surveillance in Scotland (ECOSS)** | **Regional Virus Laboratory (RVL)** |
| **Outcome** | 3.1. Notified biologically/lab-confirmed cases | Yes | Yes | No | Yes | Yes | Yes | Yes | Yes |
| **Granularity** | Age group | Unpublished | 0-4, 5-14, 15-34, 35-59, 60-79, 80+ | None or unpublished | No | No | >1, 1-4, 5-14, 15-24, 25-34, 35-44, 45-64, 65-74, 75+ | No | 0-4, 5-14, 15-64, 65+ |
|  | Gender | Unpublished | Yes |  | No | No | No | No | No |
|  | Risk condition | No | No |  | No | No | No | No | No |
|  | Location | No | Yes, per region |  | No | No | No | No | No |
|  | Virology | Flu virus type & sub-type, other viruses (RSV & others) | No |  | Flu virus type & sub-type | Flu virus type & sub-type, other viruses (RSV & others) | Flu virus type & sub-type, other viruses (RSV & others) | Yes virus type, sub-type | Flu virus type & sub-type, other viruses (RSV & others) |
|  | Severity (symptoms, complications) | No | Yes |  | No | No | No | No | No |
|  | Treatment | Unpublished | Yes (hospitalization & ventilation) |  | No | No | No | No | No |
|  | Vaccination status | Unpublished | No |  | No | No | No | No | No |
| **Timing** | Frequency | Weekly | Weekly |  | Weekly | Weekly | Weekly | Weekly | Weekly |
|  | Time period | W40-W20 | All year long |  | W40-W20 | W40-W20 | W40-W20 | Week 40-Week21 | All year long |
| **Representa-tiveness** | Geographical representativeness | National | National |  | National | National | Wales | Scotland | Northern Ireland |
|  | Population representativeness | No info | 100% / mandatory |  | No info | No info | 100% / mandatory | 100% / mandatory | 100% / mandatory |
|  | Number of settings | No info | No info |  | 20 laboratories, coordinated by the NIC Centro Nacional de Microbiología | England PHE and NHS laboratories | 2 laboratories, Swansea and Cardiff | All HPS labs | All non-sentinel virology settings |
|  | Proportion of facilities | No info | No info |  | No info | No info | No info | No info | No info |
| **Sampling Strategy** | Surveillance type | Active sentinel virological surveillance | Passive systematic notification |  | Active & Passive virological surveillance | Active & Passive virological surveillance | Passive systematic notification | Passive systematic notification | Non-sentinel virology surveillance |
|  | Sampling | From sentinel GPs (Réseau Sentinelle) & hospitals (RENAL) | From GP practice, Hospitals & others |  | From sentinel and non sentinel sources | From sentinel and non sentinel sources | From sentinel, non-sentinel GPs & all wards from hospitals | From non-sentinel sources, mostly hospitals | From GP practice, Hospitals, & others |
|  | Test type | RT-PCR & others (rapid point of care) | RT-PCR |  | RT-PCR | RT-PCR | RT-PCR | RT-PCR & RPOCT | RT-PCR |
| **Communication** | In annual report | Yes | Yes |  | Yes | Yes | Yes | Yes | Yes |
|  | In weekly report | Yes | Yes |  | Yes | Yes | Yes | Yes | Yes |
|  | Delay in release | No | No |  | No | No | No | No | No |
|  | Data can be extracted | Yes | Yes |  | Yes | Yes | Yes | No | Yes |
|  |  | Data available & published | Unpublished data | None | No info | NA |  |  |  |

**4. Outbreak surveillance**

|  |  | **France** | **Germany** | **Italy** | **Spain** | **England** | | **Wales** | **Scotland** | **Northern Ireland** |
| --- | --- | --- | --- | --- | --- | --- | --- | --- | --- | --- |
| **Surveillance tools** | | **Social and medical setting outbreak monitoring** | **Mandatory outbreak notification** | **Public setting outbreak monitoring** | **Public setting outbreak monitoring** | **Public setting outbreak monitoring** | **School surveys** | **Public setting outbreak monitoring** | **Public setting outbreak monitoring** | **Public setting outbreak monitoring** |
|  |  | **Ehpad outbreak reporting** | **IfSG obligation** | **NA** | **"Brotes" surveillance** | **Health Protection Team surveillance scheme** | **Medical Officers of Schools Association (MOSA)** | **Health Protection Team** | **Health Protection Scotland (HPS)** | **Health Protection Duty Room Services** |
| **Outcome** | 4.1. ARI / ILI outbreaks in  closed settings | Yes | No | Unpublished | Yes | Yes | ILI incidence rates | Yes | Yes | Yes |
|  | 4.2. Biologically/laboratory-confirmed outbreaks in closed settings | Yes | Yes |  | Yes | Yes | No | Yes | Yes | Yes |
| **Granularity** | Age group | No | In some cases |  | Median age only | No | Yes | No | No | No |
|  | Gender | No | Yes |  | No | No | No | No | No | No |
|  | Risk condition | No | No |  | No | No | No | No | No | No |
|  | Location | No | Yes, per region |  | Per type of institution | Institution type (care homes, hospitlas, schools, other) | No | Yes, by Institution type and Health region | Yes, by region & Institution type (care homes, hospitlas, schools, other) | Institution type (care homes, hospitals, schools, other) |
|  | Virology | Influenza or other / unknown virus type | Flu virus type & sub type |  | Flu virus type & sub type | Flu virus type & sub-type, other viruses (RSV & others) | NA | Flu virus type & sub-type, other viruses (RSV & others) | Flu virus type & sub-type, other viruses (RSV & others) | Flu virus type & sub-type, other viruses (RSV & others) |
|  | Severity (symptoms, complications) | Attack rate, Hospitalizations, deaths | Yes |  | Hospitalizations, deaths | No | No | No | No | No |
|  | Treatment | Yes | Yes |  | NA | No | No | No | No | No |
|  | Vaccination status | HCW & Patient VCR | No |  | No | No | Available | No | No | No |
| **Timing** | Frequency | Weekly | Weekly |  | Weekly | Weekly | Weekly, yearly | Weekly | Weekly | Weekly |
|  | Time period | W40-W20 | W40-W20 |  | W40-W20 | W40-W20 | Once | W40-W20 | W40-W20 | W40-W20 |
| **Representa-tiveness** | Geographical representativeness | National | National |  | National, regional | England | England | Wales | Scotland | Northern Ireland |
|  | Population representativeness | EhpaD residents | 100% / mandatory |  | 100% / mandatory | 100% / mandatory | 15k students% | 100% / mandatory | 100% / mandatory | 100% / mandatory |
|  | Type of settings | Long term care facilities (Ehpad) | Health care facilites (e.g. hospitals, nursing homes) & others |  | Nursing homes, schools / kindergarten, medical settings & others | Hospitals, care homes, schools & others | Educational settings | Hospitals, care homes, schools, nursery, prisons & others | Hospitals, care homes, schools, nursery & others | Hospitals, care homes, schools, nursery & others |
|  | Number of settings | Unpublished | No info |  | No info | No info | No info | No info | No info | No info |
|  | Proportion of facilities | 100% Ehpad | No info |  | No info | No info | No info | No info | No info | No info |
| **Sampling Strategy** | Surveillance type | Passive systematic notification | Passive systematic notification |  | Passive systematic notification | Passive systematic notification | Passive systematic notification | Passive systematic notification | Passive systematic notification | Passive systematic notification |
|  | Sampling | Occasional | Systematic |  | Systematic | No info | No | Systematic testing in case of ARI or ILI outbreak | Systematic reporting (and testing) in case of ARI outbreak | No info |
|  | Test type | RT-PCR & others (rapid point of care) | RT-PCR |  | RT-PCR | RT-PCR | No | RT-PCR | RT-PCR | RT-PCR |
| **Communication** | In annual report | Yes | Yes |  | Yes | Yes | No | Yes | Yes | Yes |
|  | In weekly report | Yes | Yes |  | Yes (daily report) | Yes | No | Yes | Yes | Yes |
|  | Delay in release | No | No |  | No | No | No | No | No | No |
|  | Data can be extracted | No | No |  | No | No | No | Yes | Yes | Yes |
|  |  | Data available & published | Unpublished data | None | No info | NA |  |  |  |  |

**5. Primary care surveillance**

|  |  | **France** | | **Germany** | | **Italy** | **Spain** | **England** | | **Wales** | | **Scotland** | | **Northern Ireland** | | |
| --- | --- | --- | --- | --- | --- | --- | --- | --- | --- | --- | --- | --- | --- | --- | --- | --- |
| **Surveillance tools** | | **Sentinel GPs** | **Sentinel GPs** | **Sentinel GPs** | **Excess GP visit modelling** | **Sentinel GPs** | **Sentinel GPs** | **Sentinel GPs** | **GPs** | **Sentinel GPs** | **GPs** | **GPs** | **Sentinel GPs** | **GPIHs** | **GPOOH** | **Sentinel GPs** |
|  |  | **Réseau Sentinelles** | **SOS Médecins** | **AGI sentinel network** | **RKI statistical modelling** | **Influnet-Vir** | **Sentinel Sistema de Vigilancia de la Gripe en España (SVGE)** | **Royal College of General Practitioners Research and Surveillance Centre (RCGP RSC)** | **GP In Hours and Out-of-Hours (GPIH & OOH)** | **GP Sentinel Surveillance of Infections Scheme** | **GP Out-of-Hours (GPOOH)** | **Scottish Influenza Surveillance Reporting Scheme (SISRS)** | **GP sentinel swabbing scheme (GPSSS)** | **NI Enhanced Surveillance of Influenza** | | |
| **Outcome** | 5.1. ARI / ILI GP visits and/or incidence rates | ARI & ILI (tele)consultations | ARI & ILI (tele)consultations | Yes | No | Yes | Collected but unpublished | Yes | Yes | Yes, ARI & ILI GP visits | Respiratory related consultations | Yes, ARI & ILI GP visits | Yes | Yes, ILI & Flu-like | Yes | Yes, ILI & Flu-like |
|  | 5.2. Biologically/laboratory-confirmed GP visits and/or incidence rates | Yes | No | Yes | No | Yes | Yes | Yes | No | Yes | No | No | Yes | No | No | Yes |
|  | 5.3. Influenza-associated excess GP visits | No | No | No | Yes | No | No | No | No | No | No | No | No | No | No | No |
|  | 5.4. Influenza-associated excess work loss cases | No | No | No | Yes | No | No | No | No | No | No | No | No | No | No | No |
| **Granularity** | Age group | 0-90, in 5-year splits | <15, 15-64, 65+ | 0-4, 5-14, 15-34, 35-59, 60+ | 0-1, 2-4, 5-14, 15-34, 35-59, 60+ | 0-4, 5-14, 15-64, 65+ | 0-4, 5-14, 15-64, 65+ | >1, 1-4, 5-14, 15-24, 25-44, 45-64, 65-74, 75-84, 85+ | <1, 1-4, 5-14, 15-44, 45-64, 65-74, 75+ | >1, 1-4, 5-14, 15-24, 25-34, 35-44, 45-64, 65-74, 75+ | No | <1, 1-15, 15-44, 45-64, 65-74, 75+ | >1, 1-4, 5-14, 15-24, 25-34, 35-44, 45-64, 65-74, 75+ | 0-4, 5-14, 15-44, 45-64, 65+ | <1, 1-4, 5-14, 15-44, 45-64, 65-74, 75+ | 0-4, 5-14, 15-44, 45-64, 65+ |
|  | Gender | Yes | No | Unpublished | No | No | No | Yes | No | No | No | Collected but unpublished | No | No | No | No |
|  | Risk condition | Per risk condition | No | No | No | Collected as Y/N but unpublished | Collected but unpublished | Yes | No | No | No | No | No | No | No | No |
|  | Location | Yes, per department | Yes, per region | Yes, per region | No | Yes, per region | Yes, per region | Yes, by PHE center | Yes, by PHE center | Yes, by Health region | No | Collected but unpublished | No | No | No | No |
|  | Virology | Flu virus type & sub-type, other viruses (RSV & others) | NA | Flu virus type & sub-type, other viruses (RSV & others) | Flu virus type, sub-type & RSV | Virus type & sub-type | Virus type & sub-type | Flu virus type & sub-type, other viruses (RSV & others) | NA | Flu virus type & sub-type, other viruses (RSV & others) | No | NA | Flu virus type & sub-type, other viruses (RSV & others) | No | NA | Flu virus type & sub-type, other viruses (RSV & others) |
|  | Severity (symptoms, complications) | Hospital admission | No | Hospital admission | NA | No | No | No | No | No | No | No | No | No | No | No |
|  | Treatment | Antiviral & antibiotic treatment | No | No (or unpublished) | NA | No (or unpublished) | No (or unpublished) | No | No | Antiviral treatment | No | No | No | No | No | No |
|  | Vaccination status | Yes | No | No | No | Collected but unpublished | Some regions | Yes | No | Collected but unpublished | No | No | Collected but unpublished | Yes | No | Collected but unpublished |
| **Timing** | Frequency | Weekly | Weekly | Weekly | Weekly | Weekly | Weekly | Weekly | Weekly | Weekly | Weekly | Weekly | Weekly | Weekly | Weekly | Weekly |
|  | Time period | W40-W20 | All year long | All year long | W40-W20 | W42-W17 | W40-W20 weekly / W20-W40 biweekly | All year long | All year long | All year long | All year long | All year long | All year long | Weeks 40-21 | Weeks 40-22 | Weeks 40-21 |
| **Representa-tiveness** | Geographical representativeness | National, regional, departmental | National & Regional | National | National | National | National | National (England), regional | England | Wales | Wales | Scotland | Scotland | Northern Ireland | England | Northern Ireland |
|  | Population representativeness | ~2% | No info | >1% | >1% | 2% | 2.4% | No info | No info | ~4000 until beginning of June | No info | ~100% population | No info | 100% | 98% | 11% |
|  | Number of settings | 1,450 GPs & paeds (300 GPs & paeds for virology) | 62 out of 63 associations | 828 GPs & paeds | NA | ~1,000 GPs | 770 GPs (incl. 210 peads) | ~360 GPs (ILI) inc ~100 GPs (virological specimens) | ~5k | ~45 GP practices, ~20 GP sending virological samples | No info | 950 GP practices | No info | 323 GP practices (in-hours) | 5 GP OOH centres (out-of hours) | 33 GP practices |
|  | Proportion of facilities | 2.3% for incidence (no info for virology) | No info | >1% (590 practices) | NA | 3% | 3% | No info |  | No info | No info | 99% | No info | No info | No info | No info |
| **Sampling Strategy** | Surveillance Type | Active sentinel syndromic surveillance | Active sentinel syndromic surveillance | Active sentinel syndromic surveillance | Excess event modelling | Active sentinel syndromic surveillance | Active sentinel syndromic surveillance | Active sentinel syndromic surveillance | Passive syndromic surveillance | Active sentinel syndromic surveillance | Passive syndromic surveillance | Passive syndromic surveillance | Active sentinel syndromic surveillance | Passive syndromic surveillance | Active sentinel syndromic surveillance | Active sentinel syndromic surveillance |
|  | Definition | ILI : >39°, myalgia and respiratory sp, sudden onset | ILI : >38,5°, myalgia and respiratory sp, sudden onset | ARI | RKI model based on AGI sentinel data | ILI | ILI | No info | ILI (in hours), ILI & ARI (out of hours) | ILI, LRTI | Respiratory related consultations | ILI (individual with ARI with physician-diagnosed fever or complaint of fever) | ILI, LRTI | ILI, Flu-like | ILI (in hours), ILI & ARI (out of hours) | ILI, LRTI |
|  | Sampling | Max 1 test / p/ week for virology | No info | All ARI patients in participating sentinel sites | NA | Random | 2 first cases of the week | Systematic | NA | No info | NA | NA | No info | NA | NA | No info |
|  | Test type | RT-PCR | No info | RT-PCR | NA | RT-PCR | RT-PCR | RT-PCR | NA | RT-PCR | NA | NA | RT-PCR | NA | NA | RT-PCR |
| **Communication** | In annual report | Yes | Yes | Yes | Yes | Yes | Yes | Yes (PHE + specific report) | Yes | Yes | No | Yes, specific report | Yes | Yes | Yes | Yes |
|  | In weekly report | Yes | Yes | Yes | No | Yes | Yes | Yes | Yes | Yes | Yes | Yes | Yes | Yes | Yes | Yes |
|  | Delay in release | No | No | No | No | No | No | Yes | No | No | No | No | No | No | No | No |
|  | Data can be extracted | No | Yes (Geodes) | No | Yes | Not all | No | Yes | No | Yes | Yes | No | No | No | No | No |
|  |  | Data available & published | Unpublished data | None | No info | NA |  |  |  |  |  |  |  |  |  |  |

**6. Hospital surveillance**

|  |  | **France** | | **Germany** | | | **Italy** | **Spain** | | **England** | | | **Wales** | **Scotland** | **Northern Ireland** | |
| --- | --- | --- | --- | --- | --- | --- | --- | --- | --- | --- | --- | --- | --- | --- | --- | --- |
| **Surveillance tools** | | **Sentinel hospitals** | **Severe outcome surveillance** | **Mandatory disease notification** | **Sentinel hospitals** | **Excess hosp. modelling** | **Severe outcome surveillance** | **Sentinel hospitals** | **Severe outcome surveillance** | **Emergency department attendances** | **Sentinel Hospitals** | **Severe outcome surveillance** | **Hospital surveillance** | **Severe outcome surveillance** | **Severe outcome surveillance** | **Severe outcome surveillance** |
|  |  | **Organisation de la surveillance coordonnée des urgences (OSCOUR)** | **Santé publique France regional teams** | **IfSG - Hospital** | **Syndromische Krankenhaus-Surveillance (ICOSARI)** | **RKI statistical modelling** | **SARI sentinel hospitals 'Casi Gravi'** | **Casos hospitalizados confirmados de gripe (CHOSP)** | **Vigilancia de casos graves hospitalizados confirmados de gripe (CGHCG)** | **Emergency Department Syndromic Surveillance System (EDSSS)** | **Severe Influenza Surveillance System (USISS) Sentinel** | **Severe Influenza Surveillance System (USISS) Mandatory** | **Hospital surveillance** | **SARI surveillance / USISS mandatory in ICUs** | **Non-ICU / HDU surveillance** | **USISS mandatory (ICU / HDU surveillance)** |
| **Outcome** | 6.1. ILI or biologically/laboratory-confirmed Emergency Department visits | Yes | No | No | No | No | Unpublished | No | No | Yes | No | No | Yes | No | No | No |
|  | 6.2. SARI / ILI hospital admissions | Yes | No | No | Yes | No | Unpublished | Yes | Yes | No | Yes | No | Yes | Yes | No | No |
|  | 6.3. Biologically/laboratory-confirmed hospital admissions | No | No | Yes | No | No | Unpublished | Yes | Yes | No | Yes | No | Yes | Yes | Yes | No |
|  | 6.4. Influenza-associated excess hospital admissions | No | No | No | No | Yes | No | No | No | No | No | No | No | No | No | No |
|  | 6.5. Biologically/laboratory-confirmed ICU admissions | No | Yes | No | No | No | Yes | Yes | Yes | No | Yes | Yes | Yes | Yes | No | Yes |
| **Granularity** | Age group | 0-4, 5-14, 15-64, 65+ | 0-4, 5-14, 15-64, 65+ | 0-1, 2-4, 5-14, 15-34, 35-59, 60-79, 80+ | 0-1, 2-4, 5-14, 15-34, 35-59, 60-79, 80+ | 0-1, 2-4, 5-14, 15-34, 35-59, 60+ | 0-4, 5-14, 14-49, 50-64, 65-74, 75+ | 0-4, 5-14, 15-64, 64+ | 0-4, 5-14, 15-44, 45-64, 64+ | Consolidated | <1, 1-4, 5-14, 15-24, 25-34, 35-44, 45-64, 65-74, 75+ | <1, 1-4, 5-14, 15-44, 45-64, 65+ | <1, 1-4, 5-14, 15-24, 25-34, 35-44, 45-64, 65-74, 75+ | <1, 1-4, 5-14, 15-44, 45-64, 65+ | No or unpublished | <1, 1-4, 5-14, 15-44, 45-64, 65+ |
|  | Gender | Unpublished | Yes | No (or unpublished) | No (or unpublished) | No (or unpublished) | No (or unpublished) | Unpublished | Yes | No | No | No | Yes | No | No | Yes |
|  | Risk condition | No | Yes, per chronic condition | No (or unpublished) | No (or unpublished) | No (or unpublished) | Consolidated | No | Yes, per chronic condition | No | No | No | No (or unpublished) | No | No | Yes |
|  | Location | Yes | No | Yes | No (or unpublished) | NA | Yes | No (or unpublished) | No (or unpublished) | No | No | per 100,000 trust catchment population for England | No (or unpublished) | Yes, by region | No | No |
|  | Virology | No | Virus type & sub-type (when available) | No | No | Flu virus type & sub-type | Virus type & sub-type | Virus type & sub-type | Virus type & sub-type | No | Virus type & sub-type | Flu virus type & sub-type, other viruses (RSV & others) | Flu virus type & sub-type, other viruses (RSV & others) | Yes, virus type, sub-type | Flu virus type & sub-type, other viruses (RSV & others) | Flu virus type & sub-type, other viruses (RSV & others) |
|  | Severity (symptoms, complications) | Severity from CCMU 1 to 5, Hospital admissions & ICU | ARDS, ventilation & deaths | Pneumonia, ECMO, ARDS & deaths | Proportion to ICU & ECMO | NA | ECMO & deaths | Proportion to ICU & deaths | Pneumonia, co-infection, SDRA, organic failure, proportion to ICU & deaths | No | Proportion to ICU, deaths | Yes, in case of admission to Severe Respiratory Failure Center | Breakdown between General in & Outpatient, Urgent care & accident / emergency wards, ICU wards | ICU admissions, Case fatality rate | No | Yes, in case of admission to Severe Respiratory Failure Center, or death |
|  | Treatment | No | No | No (or unpublished) | No (or unpublished) | NA | No (or unpublished) | No (or unpublished) | No (or unpublished) | No | No | No | No (or unpublished) | No | No | No (or unpublished) |
|  | Vaccination status | No | Yes | No | No | No | Yes | No | Yes | No | Yes | No | No (or unpublished) | No | No | Yes |
| **Timing** | Frequency | Weekly | Weekly | Weekly | Weekly | Annual | Weekly | Weekly | Weekly | Weekly | Weekly | Weekly | Weekly | Weekly | Weekly | Weekly |
|  | Time period | All year long | W45-W15 | All year long | All year long | W40-W20 | W42-W17 | All year long | All year long | All year long | All year long | All year long | All year long | Week 40-Week20 | All year long | All year long |
| **Representa-tiveness** | Geographical representativeness | National, regional | National | National | National | National | National | National, regional | National, regional | England | England | UK, England, Scotland, Northern, Ireland | Wales | Scotland | Northern Ireland | UK, Northern Ireland |
|  | Population representativeness | 93% | 60-70% | 100% | 6% | No info | 50-100% | ~52% | ~52% | No info | ~10% | 100% | No info | No info | No info | 100% |
|  | Number of settings | 690 | 192 ICU units | All medical settings | 73 | No info | No info | 100 | 100 | 25 sentinel EDs | ~44 NHS acute trusts | All ICU | No info | No info | No info | All ICU |
|  | Proportion of facilities | No info | No info | 100% | No info | No info | No info | No info | No info | All | No info | 100% | No info | No info | No info | 100% |
| **Sampling Strategy** | Surveillance Type | Active sentinel syndromic surveillance | Active sentinel syndromic surveillance | Passive systematic notification | Active sentinel syndromic surveillance | Excess event modelling | Active sentinel syndromic surveillance | Active sentinel syndromic surveillance | Active sentinel syndromic surveillance | Active sentinel syndromic surveillance | Active sentinel syndromic surveillance | Active sentinel syndromic surveillance | Active sentinel syndromic surveillance | Active sentinel syndromic surveillance | Active sentinel surveillance | Active sentinel surveillance |
|  | Definition | CIM 10 (ILI or Flu) | SARI | Lab confirmed | ICD 10 Codes J09-J22 | RKI model based on AGI Sentinellen data (very conservative as only include hospital admission after ARI consultation at GP) | SARI | Lab-confirmed, independent of severity | Lab-confirmed severe cases | ARI | ARI | SARI | ARI | SARI | All lab-confirmed influenza cases in hospitals | All lab-confirmed influenza cases in level 2 and 3 critical care units |
|  | Sampling | NA | Sentinel SARI | No info | No info | NA | No info | No info | No info | NA | No info | Systematic | No info | No info | No info | Systematic |
|  | Test type | NA | RT-PCR & others (rapid point of care) | RT-PCR & others | Multiple tests | NA | RT-PCR | RT-PCR/ viral culture / Antibody assay | RT-PCR/ viral culture / Antibody assay | NA | RT-PCR | RT-PCR | RT-PCR | RT-PCR | RT-PCR | RT-PCR |
| **Communication** | In annual report | Yes | Yes | Yes | Yes | Yes | Yes | Yes | Yes | Yes | Yes | Yes | Yes | Yes | Yes | Yes |
|  | In weekly report | Yes | Yes | Yes | Yes | No | Yes | Yes | Yes | No | Yes | Yes | Yes | Yes | Yes | Yes |
|  | Delay in release | No | No | No | No | No | No | No | No | No | No | No | No | No | No | No |
|  | Data can be extracted | Yes | No | Yes | No | No | Yes | Yes | Yes | Yes | No | No | Yes | No | No | No |
|  |  | Data available & published | Unpublished data | None | No info | NA |  |  |  |  |  |  |  |  |  |  |

**7. Mortality surveillance**

|  |  | **France** | | | | **Germany** | | | **Italy** | | **Spain** | | **England** | | | **Wales** | **Scotland** | | **Northern Ireland** | |
| --- | --- | --- | --- | --- | --- | --- | --- | --- | --- | --- | --- | --- | --- | --- | --- | --- | --- | --- | --- | --- |
| **Surveillance tools** | | **Hospital lab-confirmed mortality** | **Mandatory death notification** | **All-cause mortality** | **Excess-mortality model** | **Mandatory disease notification** | **Excess-mortality modelling** | **Excess-mortality modelling** | **Hospital lab-confirmed mortality** | **Excess-mortality modelling** | **Hospital lab-confirmed mortality** | **Excess-mortality modelling** | **Hospital lab-confirmed mortality** | **Lab-confirmed mortality** | **Excess-mortality modelling** | **Excess-mortality model** | **Hospital lab-confirmed mortality** | **Excess-mortality model** | **Death statistics** | **Excess-mortality model** |
|  |  | **Santé publique France regional teams** | **Electronic death cause registry (CépiDc)** | **Santé Publique France - INSEE** | **SpF algorithm + FluMOMO** | **IfSG Obligation** | **RKI Model** | **EuroMOMO/ FluMOMO** | **Casi gravi** | **Sismg + EuroMOMO** | **Vigilancia de casos graves hospitalizados confirmados de gripe (CGHCG)** | **MOMO** | **Severe Influenza Surveillance System (USISS) Mandatory** | **Peadiatric mortality** | **FluMOMO** | **EuroMOMO** | **SARI Surveillance** | **EuroMOMO** | **Respiratory associated deaths (NISRA)** | **EuroMOMO** |
| **Outcome** | 7.1. Diagnosed or biologically/laboratory-confirmed deaths | Yes | Yes (Flu, CIM 10) | No | No | Yes | No | No | Yes | No | Yes | No | Yes | Yes | No | No | Yes | No | Yes | No |
|  | 7.2. Influenza-associated excess deaths | No | No | Yes (all causes) | Yes (all causes & attributable to flu) | No | Yes | Yes | No | Yes | No | Yes (all causes) | No | No | Yes | Yes | No | Yes | No | Yes |
| **Granularity** | Age group | 0-4, 5-14, 15-34, 35-64, 65+ | by 5y age group | 0-64, 65-74, 75+ | 0-64, 65-74, 75+ | 0-4, 5-14, 15-34, 35-59, 60-79, 80+ | Consolidated | Consolidated | 0-4, 5-14, 14-49, 50-64, 65-74, 75+ | 65+: 65-74, 75-84, +85 | 0-4; 5-14, 15-44; 45-64, 64+ | <65, 65-74, 75+ | <1, 1-4, 5-14, 15-44, 45-64, 65+ | Consolidated | <5, 5-14, 15-64, 65+ | <5, 5-14, 15-64, 65+ | <1, 1-4, 5-14, 15-44, 45-64, 65+ | <5, 5-14, 15-64, 65+ | No | <5, 5-14, 15-64, 65+ |
|  | Gender | No | No | No | No | Yes | No | No | Yes | No | Yes | Yes | No | No | No | No | No | No | No | No |
|  | Risk condition | No | No | No | No | Unpublished | No | No | Consolidated | No | Yes, per chronic condition | No | No (or unpublished) | Yes | No | No | No | No | No | No |
|  | Location | No | No | National/regional | National/regional | Yes | No | Yes (Berlin & Hesse) | Yes, per region | No | No | National, regional | unpublished | No | No | No | Yes, by region | No | No | No |
|  | Virology | Virus type & sub-type | No | No | No | Virus type & sub-type | No | No | Virus type & sub-type | No | Virus type & sub-type | No | Virus type & sub-type | Virus type & sub-type | No | No | Yes virus type, sub-type | No | No | No |
|  | Severity (symptoms, complications) | NA | NA | NA | NA | NA | NA | NA | NA | NA | NA | NA | NA | NA | NA | NA | NA | NA | NA | NA |
|  | Treatment | NA | NA | NA | NA | NA | NA | NA | NA | NA | NA | NA | NA | NA | NA | NA | NA | NA | NA | NA |
|  | Vaccination status | Yes | NA | NA | NA | No | NA | NA | Yes | NA | Yes | NA | No (or unpublished) | Yes | NA | NA | No | NA | NA | NA |
| **Timing** | Frequency | Weekly | Weekly | Weekly | Weekly | Weekly | Annual | Annual | Weekly | Annual | Weekly | Weekly | Daily | Annual | Weekly | Weekly | Weekly | Weekly | Weekly | Weekly |
|  | Time period | All year long | All year long | All year long | All year long | All year long | All year long | All year long | W42-W17 | All year long | All year long | All year long | All year long | All year long | All year long | All year long | Week 40-Week20 | All year long | All year long | All year long |
| **Representa-tiveness** | Geographical representativeness | National | National/regional | National/regional | National/regional | National | National | Regional | National | National | Wk 40 -Wk 20 | National, regional | England | England | England | Wales | Scotland | Scotland | Northern Ireland | Northern Ireland |
|  | Population representativeness | No info | ~20% | 77% national deaths | 77% national deaths | 100% | 100% | Berlin & Hesse only | 50-100% | 20% | 52% | 93% | 100% | 100% | 85% | No info | No info | No info | No info | No info |
|  | Number of settings | 192 ICU units | All settings | 3000 municipalities | 3000 municipalities | All medical institutions | No info | No info | No info | No info | 100 | 3929 civil registries | 132 | No info | No info | No info | No info | No info | No info | No info |
|  | Proportion of facilities | Out of 376 ICUs | NA | No info | No info | 100% | NA | NA | Not all ICU units | No info | No info | All provinces | 92% | No info | No info | No info | No info | No info | No info | No info |
| **Sampling Strategy** | Surveillance type | Active sentinel surveillance | Death Registry | Passive systematic notification | Excess death modelling | Passive systematic notification | Excess death modelling | Excess death modelling | Active sentinel surveillance | Excess death modelling | Active sentinel surveillance | Excess death modelling | Active sentinel surveillance | Passive systematic notification | Excess death modelling | Excess death modelling | Active sentinel syndromic surveillance | Excess death modelling | Death certificate analysis | Excess death modelling |
|  | Definition | SARI | Attributable to influenza | All causes | All causes & attributable to influenza | Lab-confirmed flu death notification | Attributable to influenza (based on all causes) | Winter excess mortality and attributable to influenza (based on all causes) | SARI | All causes | No info | All causes | SARI | Lab-confirmed flu death notification | All causes & attributable to influenza | All causes | SARI | All causes | Respiratory associated deaths include those that are attributable to influenza, other respiratory infections or their complications. This includes “bronchiolitis, bronchitis, influenza or pneumonia” keywords recorded on the death certificate. | All causes |
|  | Test type | RT-PCR | No info | NA | NA | Multiple tests | NA | NA | RT-PCR | NA | RT-PCR/ viral culture / Antibody assay | NA | RT-PCR | RT-PCR | NA | NA | RT-PCR | NA | NA | NA |
| **Communication** | In annual report | Yes | No | Yes | Yes | Yes | Yes | Yes | Yes | Yes | Yes | Yes | Yes | Yes | Yes | Yes | Yes | Yes | No | Yes |
|  | In weekly report | Yes | No | Yes | Yes | No | No | No | Yes | No | Yes | Yes, specific report | Yes | No | Yes | Yes | Yes | Yes | Yes | Yes |
|  | Delay in release | No | Yes (2 years) | No | No | No | Yes | No | No | No | No | No | No | No | No | No | Yes | No | No | No |
|  | Data can be extracted | No | No | No | No | Yes | No | No | Yes | Not all | Yes | No | No | No | Yes | No | No | Yes | No | Yes |
|  |  | Data available & published | Unpublished data | None | No info | NA |  |  |  |  |  |  |  |  |  |  |  |  |  |  |
